# Program Quality and Developmental Outcomes Related to Youth Volleyball in Ethiopia: Assessing Relationships and Variations

**DOI:** 10.3390/ejihpe11040100

**Published:** 2021-11-08

**Authors:** Abdulaziz Mussema, Tefera Tadesse, Zelalem Melkamu

**Affiliations:** 1 Department of Sports Sciences, College of Natural and Computational Sciences, Wolkite University, Wolkite City P.O. Box 07, Ethiopia; 2 Institute of Educational Research, Addis Ababa University, Addis Ababa City P.O. Box 1176, Ethiopia; 3 Sport Academy, Bahir Dar University, Bahir Dar City P.O. Box 79, Ethiopia

**Keywords:** youth sport, program quality, positive youth development, quantitative methods

## Abstract

This study aims to assess program quality and developmental outcomes of a youth volleyball project in one of the regional states in Ethiopia, and further examine variations between groups across gender and project site zones. We applied a cross-sectional survey design, collecting quantitative data from youth volleyball players (*n* = 215) with a mean age of 16.18 years (SD = 0.69) through a self-reported questionnaire. The results indicated that young players’ perceptions did not vary significantly across gender, except for the mean score of the perceived experience variable for girls (M = 2.68, SD = 0.318) was significantly higher than the mean score of boys (M = 2.58, SD = 0.258). One-way (project site zone) analyses of variance (ANOVAs) identified that youth volleyball projects in the central zone were consistently rated higher than those in the western zone, except for the current practice rating. Moreover, correlation analysis results indicated the presence of a significant relationship, both within and between program quality and developmental outcome variables. Furthermore, the results of regression analysis indicated that the program quality variables together predicted each of the developmental outcomes, accounting for 18.9% to 31.7% of the variances. It is concluded that the quality of the youth volleyball program in Ethiopia varies considerably across the project site zones and the program quality variables significantly relate to the developmental outcomes measured with differential effects. The data from this study reveals several practical applications for Ethiopia and beyond in terms of guiding youth volleyball projects. Moreover, the findings of the study showed that youth sport and the manner in which it is structured and delivered to youth players influences the attainment of positive developmental outcomes. These results suggest that contextual differences really do have an effect on the quality of youth sport program processes and developmental outcomes.

## 1. Introduction

### 1.1. Background

Sport for Development (SFD) is a growing research field that uses sports to achieve key outcomes for youth, such as learning, health, empowerment, and protection, among others [1,2]. Positive youth development (PYD) interventions are widely used to prevent youth substance use and violence [3]. Sports participation can have a significant positive effect on PYD as it enhances personal development not only in physical but also psychosocial domains [4,5]. Indeed, one of the most important benefits of using sports intervention is the ability to teach life skills that help individuals to be equipped with the tools for handling difficult life situations so they learn to develop more positive perspectives for their own futures [6]. Hence, youth sports programs are essential mechanisms for PYD, as they offer multiple benefits [7].

Youth program quality refers to the structure and processes within a program that is intentionally designed and implemented to promote PYD outcomes [8]. Researchers argue that program quality, encompassing essential functions, behaviors, and actions, is the best predictor of positive developmental outcomes [9]. Using program quality is recommended when evaluating sports programs to ensure that youth who participate in a program achieve positive developmental outcomes [10,11]. Thus, using program quality in studies to assess outcomes associated with participation in youth development programs is an important exercise [12]. Various process features are identified by researchers as determinants of PYD outcomes, including opportunities to belong, positive social norms, and supportive relationships, among others [13]. It is also important that a suitable training environment, opportunities for broader physical, personal, social skill development, and the presence of supportive interactions are evident [14].

The literature review shows that program quality can be assessed in different ways, including the use of qualitative methods, quantitative self-report measures, and observational measures [15]. While qualitative methods such as a case study or a phenomenological study enable the study of a phenomenon in-depth [16], observational measures of program quality allow for the description of behavior in natural environments [17,18]. In youth sport research, most studies have relied on self-report measures [19,20], and this study used the same quantitative self-reported measures from the youth players who participated in the youth volleyball project studied. These measures were selected because they help us to understand if the youth volleyball program—that was the focus of this research—provided a climate that promotes PYD based on the participating youths’ perceptions [2].

Participation in youth sport has been associated with improved physical and psychosocial development [21,22] and other sports-based developments [23]. To best understand the outcomes emanating from youth sport, there is a need to examine the processes associated with the developmental outcomes that can be achieved [24]. The preponderance of evidence indicates that engagement in youth sport plays an important positive role in youth personal and life skills development [4,25]. Furthermore, youth players’ positive perception about their coach, as well as his or her meaningful sport lessons, leads to reported greater development in emotional regulation and cognitive skills [26]. A positive relationship is described as ‘‘the need to feel belongingness and connectedness with others’’ [27] p. 68. Youth athletes in high-quality programs perceived significantly greater opportunities for relatedness, as well as lower negative experiences [28]. Hence, coaching actions and social climates have an important influence on the personal and social development of young people [25]. The social support received from head coaches predicted athletes’ satisfaction with coaches and sport experiences, leading to success in sports [29,30].

In the present study, developmental outcome refers to the psychosocial developments attained by the athlete and further developments in sports as a result of youth involvement in organized sports [31]. The growing number of sport-based youth development programs provide a potential avenue for integrating sport meaningfully into a development agenda [19]. As a result, program quality has been outlined as one of the predictors of the developmental outcomes resulting from participation in youth programs [12]. Differences in the quality of youth sports programs may help to explain variations or differences in the sport development outcomes [32,33]

There are various outcomes of program quality in youth sports. One way of measuring it is using the impact of youth sport on health and positive youth development (PYD) [34,35]. The other one is using sporting success explained through widening participation and promoting sporting excellence [36,37]. Additionally, the role that the sport experience plays in the development of positive personal and life skills in youth is also recognized [38,39]. In the current study, the authors used a hybrid of quality measures, including youth volleyball participants’ perceived quality of talent identification and the development of a system for recognizing talent, the extent of participation in youth volleyball, an integrated system for youth volleyball, and national and regional volleyball competitions [15,19]. In a complex setting such as youth volleyball, different program qualities interact to produce positive outcomes for the youth [7]. In fact, several factors such as program structure and processes, as well as the relationship between the athlete and the coach, contribute to making sport a place where quality youth development can occur [23].

Drawing upon the essence of SFD and PYD, the hypothesis of this study is that among youth volleyball players, positive developmental outcomes will be influenced by youth sports program quality variables such as (perceived practice, player’s experience, challenges, and coach–athlete relationship). Differences in program quality related to youth volleyball may help to explain variations in the developmental outcomes of youth volleyball participation [28]. Accordingly, it is hypothesized that demographic and contextual differences may affect program quality and developmental outcomes related to youth volleyball participation.

In Ethiopia, as in many African countries, football is the most popular sport, while long-distance running is the most productive sport [40]. It has been a long tradition for Ethiopia to win medals in the International World Championship and Olympic competitions. Ethiopian runners have dominated the middle- and long-distance events in athletics and have persistently demonstrated athletics excellence in international cross-country and road-racing competitions as well [41]. Youth Athletics, Volleyball, Basketball, and Handball are among the second most popular sports next to Football. Youth volleyball participation currently includes a large number of youth participants in schools and youth volleyball projects in the different regions of the country.

With the intent to comprehensively assess the state of quality youth volleyball, this study identified four domains of program quality that are associated with the developmental outcomes of participation in youth sports [7,38]. These are perceived practice, the player’s experience, the challenges that are confronted, and the athlete-coach relationship.

### 1.2. Statement of the Problem

PYD [42] and SFD [43] are important lines of investigation for youth sports program quality [44]. However, little evidence exists that supports youth program benefits [45,46]. Moreover, in a broader review of SDP, football was identified as the most commonly used sport in SDP programs followed by basketball and rugby [47]. Hence, it is imperative to recognize this shortcoming [48]. Moreover, there is a growing interest in defining what high-quality youth volleyball programs look like, how they can be measured accurately, and how the evidence collected can be used to improve program quality [49].

In recent years, increased availability of youth access to sport-based development programs has led to greater expectations for the quality of such programs. Research on the influence of youth sports programs on youth development is minimal and most often tends to report in the context of developed countries [10]. Moreover, while sports-based interventions were found relevant in preventing adolescent substance use and youth violence, empirical evidence supporting these interventions is scanty [3,50]. Therefore, the aim of this study was to test the relationship of four selected program quality variables and another four developmental outcome variables related to youth volleyball in Ethiopia and further identify demographic and contextual differences. More specifically, the study answered the following basic research questions.

### 1.3. Basic Research Questions

1. What is the current state of youth development programs as demonstrated in the program quality processes and their multiple outcomes in the Ethiopian context?
2. Do youth volleyball players’ perceptions of program quality and developmental outcomes vary across gender and project site zones in the Ethiopian context?
3. Do youth volleyball players’ current practice, perceived experiences, perceived challenges, and coach–athlete relationships associate with the quality of youth volleyball development in the Ethiopian context?
4. Do current practices, perceived experience, perceived challenges, and a positive coach–athlete relationship predict the quality of youth volleyball sport-based development in the Ethiopian context?

### 1.4. The Study Context

The notion of SFD emerges as a key strategic direction to use the untapped potential of sports in safeguarding the health of the nation, at the same time, equipping young players with athletic skills. One important element of such a notion is youth volleyball development. This paper follows this line of research to examine the elements of a youth volleyball project designed in one of the regional states in Ethiopia. The reason for this is that the structure, context, and delivery of youth sports programs are important in supporting or hindering positive developmental outcomes [12]. Hence, evidence obtained from this line of research greatly increases our understanding of how youth programs and practices with youth can impede or enhance their development. In Ethiopia, youth sports projects are delivered to children in collaboration with many stakeholders. Youth sports projects are devised to produce talented young male and female athletes to participate in national and international competitions representing their country [51]. These projects are operating in an equitable environment where boys and girls had equal opportunities to succeed.

## 2. Materials and Methods

### 2.1. Study Design

This study used a cross-sectional survey design. This design is helpful to gather quantitative data, such as scores on instruments that produce specific numbers that can be statistically analyzed, as it can yield results to assess the relationship between variables and measure prevalence for the variables of interest [52]. The independent variable in the analysis was the program quality components, which consisted of four domains: current practice in youth volleyball, perceived experience in youth volleyball, perceived challenges in youth volleyball, and coach–athlete relationship in volleyball. The dependent variables included quality of talent identification and development system, youth volleyball sport participation, integrated system for youth volleyball, and national and regional volleyball competitions.

### 2.2. Population, Sample Size, and Sampling Techniques

Ethiopia consists of regional states and special city administrations, and these are divided into zones, depending on geographic proximity. Hence, zones are the second level of the administration. Each zone consists of districts of Ethiopia that are known as Woredas, and these are the third level of the administration of Ethiopia—next to zones and regional states. The target population of this study encompasses all under 17 youth volleyball players in Ethiopia (between the age of 15 and 17)—(*n* = 13,200).

Each Regional state consisted of 10 youth volleyball development projects (both male and female youth players) found in 10 different youth volleyball development facilities. Each youth volleyball development project facility had a total of 120 youth players containing 60 (girls) and 60 (boys); therefore, 1200 youth volleyball players were involved in each region’s U-17 youth volleyball training development projects.

Sample selection followed a multistage sampling procedure, dividing the population into smaller and smaller groupings to create a manageable sample. First, one Regional State, that is, Southern Nations Nationalities, Peoples’ Regional (SNNPR) State was selected using a simple random sampling method. Following that, the 10 zones in the selected Regional State were purposefully considered as the 10 youth volleyball sites as they were located in these zones. Finally, 20% of the sample youth volleyball players were selected from each volleyball project. The youth players sample (*n* = 22, girls = 11, and boys = 11) in each study site was approached, and a total of 220 participants were involved in the study. To identify the youth player sample participants in each project site, the principal author obtained the list of youth players’ names from each youth volleyball development facility head. Then, sample participants (*n* = 22, girls = 11, and boys = 11) were selected using a simple random sampling method with two categories of girl and boy groups. Then, each questionnaire was administered to each sample participant. Eligible participants were youth volleyball players (*n* = 220, girls = 110, and boys = 110), each of whom had an active engagement in the youth volleyball project in each sample youth and sports development facility during the 2019 project implementation season as youth players.

The project studied is the training arm of the sports institutions as it runs training, learning, and capacity development services for youth volleyball players. The average age of the youth participants was 16.18 years (SD = 0.69). Youth volleyball projects are located in three geographical divisions called zones. These include: western zone (Konta, Segen, & Dawaro, SNNPR, Ethiopia), central zone (Wolita, Kanbata, Hadiya & Gedio, SNNPR, Ethiopia), and eastern zone (Sidama, Gamogofa, & South Omo, SNNPR, Ethiopia) (2019). Table 1 presents the summary of the demographic characteristics of the study participants (2019).

The study sample involved 107 males (49.8%) and 108 females (50.2%) with a mean age of 15.81 years (SD +0.76) for females and a mean age of 16.54 years (SD +0.62) for males. Attrition rates were low with 69.3% attending for four years (69.3%) and 30.7% attending three years. Youth volleyball players involved in this study were distributed across the three divisions, with the western zone accounting for 40.5%, the central zone 29.8%, and the eastern zone 29.8%. All had 3 to 4 years of playing experience.

### 2.3. Study Variables

Again, this comprehensive assessment included a program quality measure of the process where the object of measurement is the program [28]. Hence, the program quality measures emphasized perceived experiences and social processes or interactions between people within the program [32]. The developmental outcome measures were designed to provide a framework of essential measures of sport-based development outcomes [53]. The specific indicators of each domain/component are drawn from youth sport program practices and research [54], and while not exhaustive, they represent concrete ways that characterize a youth sports program and its impacts on development. Table 1 presents the categorization of variables included in the study.

The majority of items used in the indicators of program quality helped to assess promotive interactions between and among youth players, and their coaches, and the extent to which young volleyball players are engaged in the program-representing participant perceptions of experiences in youth programs. However, the program quality indicators also addressed the perceived challenges and the coach–athlete relationships within the program. The other domains/components of developmental outcomes focus on four major elements of developmental outcomes: Perceived quality of talent identification and development system, participation in youth volleyball, integrated system for youth volleyball, and national and regional volleyball competitions (Table 2).

### 2.4. Data Collection Instrument

In this study, the authors used a questionnaire to collect data from youth volleyball players. In order to clarify the factors and developmental outcome measures, Table 3 illustrates the definition of each measure and its sources.

The validity and reliability of the items were checked by expert review and a pilot test with participants from similar youth volleyball projects. Item-to-total correlations were assessed, as well as additional confirmatory factor analysis, which suggested a model of good-fit and constructs validity of the scale. For reliability, the internal consistency coefficients ranged from 0.81 to 0.87, which satisfies the criteria outlined in sports sciences research [18]. Each item in the questionnaire was measured using a five-point Likert scale, ranging from 1 (strongly disagree) to 5 (strongly agree). Furthermore, demographic information was collected to compare responses between subgroups. Level of training experience, youth volleyball development facility locations and low achievers), and gender were the primary areas of interest.

### 2.5. Data Collection Procedure

The principal author collected the relevant data from each study site beginning from 10 November to 30 December 2019. To do so, the principal author first obtained permission from the respective youth and sports authorities at three levels: The Southern Nation and Nationalities Peoples’ Regional (SNNPR) State, Zone, and Woreda.

Moreover, the permission included each youth volleyball development facility head to ensure their cooperation in the study. Besides cooperation, their permission also acknowledges that they understand the purpose and ethicality of the study. Additionally, the authors asked for parental/guardian consent for the youth players’ voluntary participation in the research study. Moreover, respondents were given a clear description of the purpose, scope, and intended outcomes of the research. The type of information required for the research was clearly stated, as in the policy for anonymity and confidentiality. Ethical clearance was sought for the study from Bahir Dar University Sport Academy Ethical Review Committee (S/A/D 6974/11) to ensure that the study did not involve questions that were offensive or personal in nature and there were no identifiable risks to the respondents’ health. The survey questionnaire data were completed by the youth player samples participants (*n* = 220, girls = 120, and boys = 120) from the 10 project sites. A total of 215 participants returned the questionnaires. The final sample included 215 participants, reflecting a response rate of 97.7%.

### 2.6. Data Analysis

Data were analyzed using IBM SPSS Statistics, Version 23.0, and the collected data were analyzed at four levels. First, descriptive statistics were used for the subscales of the program quality and reported developmental outcome measures to understand the perceived state of these measures in the studied context (Research question 1). Then, t-tests and one-way ANOVAs were calculated on the dependent and independent variables to examine the pattern of significant differences between sample youth volleyball players classified by their gender and project site zone (Research question 2). Significant differences between groups were determined by *p*-values ≤ 0.05. Effect sizes are important because whilst the independent t-test or ANOVA tells us whether differences between group means are “real,” it does not tell us the “size” of the difference. Effect sizes were computed to overcome this limitation and measure the magnitude of differences [57]. The most popular effect size measure is Cohen’s d [58]. In this study, the authors used an online calculator to compute the different effect sizes using Cohen’s d and eta squared [59]. The results are interpreted in the discussion section.

Then, two sets of analyses were used to examine the relationship between the quality of youth volleyball outcomes and the process correlates, including participation, experience, challenges, and coach–athlete relationship (Research question 3). The first set of bivariate correlations were calculated between the total scores of the eight self-report measures as a preliminary step to examine the relationship between the process and outcome variables of interest. The second set used multiple regressions to assess the relative influence of the four program quality variables in predicting the quality of youth volleyball outcomes.

### 2.7. Preliminary Analysis

The authors conducted preliminary analyses to ensure no violation of the assumptions of normality, linearity, multicollinearity, and homoscedasticity. A histogram was used to test the normality distribution of residuals. The result indicated that the majority of the scores lie around the center of the distribution; in addition, the coefficient of the skewness data value is between −3 and 3 and the kurtosis value is not far from zero. Thus, it fulfills the assumption of multiple regressions.

A normal probability plot or normal quantile plots of the residuals are an indicator for best tests for normally distributed errors [57]. In this study context, the points on a plot fall close to the diagonal line. This result showed that the distribution is linear. Hence, it fulfills the assumption of multiple regressions.

In examining the correlation matrix of independent variables, none of the pair of correlation coefficients exceeded 0.86 [60]. Similarly, the results revealed that no tolerance value found below 0.1 and all-variable inflation factors (VIF) values are below 10 (the VIF current practice of youth volleyball, perceived experience in youth volleyball, perceived challenges in youth volleyball, and coach–athlete relationship in youth volleyball is found below 10). Based on this, multicollinearity was not a problem in this study context [57].

## 3. Results

The results showed that the nature of the variables under examination, the existing group variations and which variables are significantly related to the quality of youth volleyball, and to what degree the program quality indicators relate to one another. The results are presented in different headings, namely, demographic statistics of participants, variations across gender and project site zones, relationship between program quality and developmental outcomes with one another, and the predictive capacity of the program quality indicators.

Overall, the ratings of program quality and developmental outcome measures were compared across gender. According to the results of this study, program quality and developmental outcomes did not vary significantly across gender, except for perceived experience, which was rated higher by female volleyball players than their male counter parts. Table 4 presents the summary of the independent *t*-test result.

As shown in Table 4, the female volleyball players’ group perceived significantly higher (M = 2.68, SD = 0.258) compared to the male volleyball players’ group (M = 2.58, SD = 0.318) in terms of perceived experience. The mean difference is 0.35 SD, which indicates a medium effect size [58].

In this study, the authors used descriptive statistics to measure the extent of positive participation, relatedness, and quality youth volleyball developmental outcomes, and provide summaries of the sample and the measures used. Table 5 presents the descriptive statistics of the variables studied.

As shown in Table 5, the mean score values for the total sample ranges between 2.53 to 3.39 with the standard deviation ranging between 0.293 to 0.818. In terms of the score distributions, for each project site division, the values ranged between mean values of 2.38 to 3.68 and the standard deviations ranged between 0.248 to 0.948. It was clear from the results of Table 5 that the ratings across the different project site zones varied considerably. However, it was unclear whether those differences were statistically significant differences or not. For this, the authors used one-way ANOVA.

One-way ANOVAs were performed to compare the effects of project site location difference on the program quality and developmental outcome variables. More specifically, there were statistically significant differences between project site zones at the *p* < 0.05 level for the current practice in youth volleyball (F (2, 212) = 21.41, *p* = 0.000, *η*^2^ = 0.16), perceived experience in youth volleyball (F (2, 212) = 15.79, *p* = 0.000, *η*^2^ = 0.12), perceived challenges in youth volleyball (F (2, 212) = 5.84, *p* = 0.003, *η*^2^ = 0.05), and coach–athlete relationship in youth volleyball (F (2, 212) = 8.22, *p* = 0.000, *η*^2^ = 0.07). Table 6 presents the summary of the one-way ANOVAs for the process quality variables studied.

According to the results in Table 6, there was a statistically significant difference between the three groups in terms of the four program quality domains related to the youth volleyball program quality measures as demonstrated by one-way ANOVAs, current practice (F (2, 212) = 21.41, *p* < 0.001), perceived experiences, (F (2, 212) = 15.79, *p* < 0.001), perceived challenges (F (2, 212) = 5.84, *p* = 0.003), and coach–athlete relationship (F (2, 212) = 8.22, *p* < 0.001). The results of the ANOVAs for the three groups (*p* > 0.131) in terms of the four developmental outcome measures were non-significant.

In order to identify which group is different from the other groups, the authors ran post hoc tests. Depending on the results of the Levin test of homogeneity of variance, the authors carried out a Tukey post-hoc comparison test when the Levin test results were not significant. Contrary to this, the authors carried out the Games Howell post-hoc test instead of Tukey’s when the Levin test results were significant. Table 7 presents the summary of the post-hoc tests.

As shown in Table 7, in three of four variables of program quality, youth volleyball projects found in the central zone were rated significantly higher than those in the western zone: Perceived experiences (*M* = 2.68 versus 2.56), Perceived challenge (*M* = 3.50 versus 3.17), and coach–athlete relationship (*M* = 3.43 versus 2.94). Also, the mean score of players’ ratings of youth volleyball projects in the central zone was significantly higher than those projects in the eastern zone. Likewise, the mean score of players’ ratings of youth volleyball projects in the eastern zone was significantly higher than the western zone in terms of the perceived challenge (*M* = 3.48 versus 3.17). However, the mean score of players’ ratings of the projects in the central zone was significantly lower than the western and eastern zones only in terms of the perceived challenge (*M* = 2.38 versus 2.64 and 2.60). Except for the current practice, a consistent perception difference was present between youth project players in the central zone and those youth project players in the western zone. This means the youth volleyball projects in the central zone were rated significantly higher than those in the western zone. taken together, these results suggest that project site location differences really do matter in program quality and attaining developmental outcomes.

For establishing relationships between constructs, and to answer the second basic research question and determine individual associations between these variables, Pearson correlation matrices were conducted for the total sample (*n* = 215). Correlations were computed among four program quality variables and another four developmental outcome variables on the data for sample (*n* = 215) participants. Table 8 presents a summary of total intercorrelations for the sample.

As shown in Table 8, the results indicated that 21 out of 28 correlations were statistically significant (16 of them had significant positive correlations and five of them had significant negative correlations). In terms of positive correlation results, 16 out of 21 correlations were statistically positively significant and were greater or equal to *r*(215) = 0.18, *p* < 0.05, two-tailed. The correlations of the perceived experience variable with the other process quality and developmental outcome variables were not statistically significant, with the exception of current practice rating, *r*(215) = 0.18, *p* < 0.01, two-tailed.

In terms of negative correlations, 5 out of 7 correlations were statistically negatively significant and were greater or equal to *r*(215) = −0.14, *p* < 0.05. Accordingly, the correlations of study participants’ ratings of perceived challenge with the other program quality and developmental outcome variables were statistically negatively significant and were greater or equal to *r*(215) ≥ −0.14, *p* < 0.05. The correlations of the perceived challenge rating with the other developmental outcome variables such as perceived experience and coach–athlete relationship were not significant, *r*(215) = −0.03, *p* > 0.05.

The third research question of this study was to investigate the predictive capacity of the four-program quality indicators on the predictions of the four developmental outcomes. For this, the authors conducted four multiple regression models. This approach was used to determine the unique variance in the dependent variable (i.e., quality of youth volleyball) that each of the independent variables explains.

The contribution of each independent variable to the variance of the dependent variable was calculated and the coefficient of determination, which is the proportion of variance in the dependent variable, that is, each developmental outcome indicator that can be explained by the independent variables was calculated. Predictors included: Current practice, player’s experience, perceived challenges, and coach–athlete relationship in youth volleyball. Table 9 presents the results of the regression models.

As shown in Table 9, in the first regression model, the four selected independent variables together predicted the quality of talent identification and development outcome, explaining 29.4% of the variance. In this model, the beta values indicated that the current practice and coach–athlete relationship in youth volleyball were found significant predictors at a comparably equal magnitude, that is, current practice (*β* = 0.28, *p* < 0.004), and coach–athlete relationship (*β* = 0.282, *p* < 0.003).

Again, in the second regression model, the four program quality indicators, together predicted the youth volleyball participation outcome, accounting for 24.7% of the total variance (R^2^ = 0.25, F(4, 210) = 17.18, *p* < 0.000). Furthermore, the effect of current practice and coach–athlete relationship to the volleyball sport participation significantly predicted youth volleyball sport participation (*β* = 0.25, *p* < 0.013), and (*β* = 0.26, *p* < 0.008).

In the third regression model, the four program quality indicators, together predicted the integrated system in youth volleyball outcome, accounting for 31.8% of the variance explained (R^2^ = 0.32, F(4, 210) = 24.47, *p* < 0.000). Furthermore, the effect of current practice and coach–athlete relationship on the integrated system in youth volleyball were found significant (*β* = 0.23, *p* < 0.016), and (*β* = 0.33, *p* < 0.001).

In the fourth regression model, the four selected independent variables collectively explained 18.9% of the total variances in national and regional sport competition outcome (R^2^ = 0.18, F(4, 210) = 12.21, *p* < 0.000), as P is less than 0.05 and F value is large, the model is significant. Furthermore, it was found that the direct effect of the variables on the national and regional volleyball sport competition was determined using a beta coefficient. Perceived challenges in youth volleyball significantly negatively predicted (*β* = −0.17, *p* < 0.008), the national and regional competition. Also, coach–athlete relationship significantly positively predicted the national and regional sport competition (*β* = 0.31, *p* < 0.003).

In general, these results of regression analyses indicate that differences in youth sports program quality, particularly current practices, perceived challenges, and coach–athlete relationships, may help to explain variations in the sport development outcomes. The persistent positive influence of coach–athlete relationship variable, which was examined as a predictor of the four developmental outcomes, further signals the relevance of the coach–athlete relationship variable in youth sport research.

## 4. Discussion

### 4.1. Youth Program Quality Processes and Outcomes

The young volleyball player samples reported a relatively higher level of coach–athlete relationship and perceived challenges, while they felt lower levels of current practice and perceived experience in youth volleyball. Girls scored significantly higher than boys on perceived experience in youth volleyball. Also, contextual differences have been observed based on the geographic location of the volleyball development facilities, youth participants’ samples in some locations perceived significantly higher program quality processes and developmental outcomes than others.

The findings of this study as well as others confirm that boys and girls accrue similar levels of physical activity during participation in youth sports [20]. Evidenced from childhood and beyond, a longitudinal study that utilized a cohort sequential design discovered that the relationships between participation and motivational beliefs did not vary significantly based on gender [33]. Hence, youth sport participation in this study context, as well as other similar contexts seems gender-neutral. However, the existence of differences between the perceived experience of female youth volleyball players and their male youth counterparts may need further investigation. When the effect sizes reported in Table 4 were compared against the commonly accepted benchmarks, the result is a small difference [58]. Similarly, the magnitude of differences for the project site zone was calculated using the formula for Eta squared [59]. All differences found in the ANOVA tests constituted an intermediate to large effect [58].

Previous research reveals contextual differences in terms of youth development and program quality [50]. We believe the findings of the group difference tests in this study complement this assertion. While comparison of the results of perceived challenges was not possible due to the absence of a similar quantitative study, results reported in qualitative studies of the same context confirmed that challenges are apparent in youth sports practices in the context of Ethiopia in particular [4], and low-income countries in general [31].

In line with this, Jowett [30] stated that the quality of the relationship among coaches and athletes may facilitate positive developmental experiences. For instance, Gano-Overway, Newton [5] found that a significant positive relationship existed between youth perceptions of a caring climate and increased prosocial behaviors. Moreover, the coach–athlete relationship has a moderate positive correlation with the developmental experiences of young athletes, accounting for a positive rating of the youth sports developmental outcome [26]. The findings of this study are consistent with these results.

Conversely, the results of correlation analysis demonstrated that there was a statistically negative significant correlation between current practice in youth volleyball and perceived challenges in youth volleyball. Also, the correlation results demonstrated that there was a statistically positive significant correlation between current practice in youth volleyball and players’ perceived experience. Supporting this, Cairney, Clark [34] stated that positive connections with teammates and stakeholders in sport are associated with good practice and positive experience, including perceived physical competence and personal and social skills.

There is also a significant relationship between program quality and developmental outcome measures. Supporting this, physical activity and sport participation represent an important component of, and contributor to, holistic quality of sports success [47]. In line with this, Silliman and Schumm [19] asserted that youth participation in a sport situation by itself does not guarantee a positive outcome, but the nature and quality of the program, which are directly dependent on input and processes, are major factors in determining benefits in youth sport. In line with this, national youth sports systems can help to clearly prioritize implementation practice and allow identification of specific areas for further improvement and gain effective outcomes [10]. In general, the correlation analyses results suggest that youth volleyball players who hold positive perceptions in one area tend to hold positive perceptions in other areas, with the exception of the perceived challenge ratings [22].

Furthermore, the regression findings reported in this study, as well as others, illustrate the complex ways in which the different program quality indicators interact to produce developmental outcomes [7]. Regardless of the difference in program design, sport-based development is theorized as a key aspect of achieving meaningful outcomes [34,48]. In this study context, this could mean paying attention to diverse sport-based development at various levels. Recent findings further support the salient relationship between the various program quality indicators and developmental outcomes. This relationship is imperative when examining such a youth volleyball project since a quality youth volleyball program can play a crucial role in promoting numerous developmental outcomes [2].

### 4.2. Limitations of the Study

Although the results of this study make a novel contribution to the literature, it is not without its limitations. First, the sample included only Under-17 (U-17) youth volleyball players and the results are more generalizable to this specific age group than others. Hence, further research is needed expanding the age cohorts to include other youth sport participants at different age levels (i.e., U-13, U-15, individual, and other team sports) than the age groups included in this study to better understand the developmental factors. Second, the selection of the study sites within one regional state in Ethiopia, that is, SNNPR, was a limitation as it does not portray the broader perspective of youth volleyball projects across Ethiopia. Third, while there are several input and program quality factors accounting for the quality of youth volleyball projects, considering only four program quality factors can be considered as another limitation of this study. Fourth, procedural steps were taken to minimize the risk of self-report bias, but even though the tools used to gather data from the questionnaire items were commonly accepted in terms of validity and reliability, the results need to be compared to other alternative measures for cross-validation. Despite these limitations, the findings provide insights on the nature of youth sports in the Ethiopian context and thus, contribute to the development of coaching theories and practices alike.

### 4.3. Practical Implications

Indeed, it is important to examine the program quality of youth sports when evaluating sports programs to understand if and how youth participants are experiencing PYD. The current study findings provide important insight on how to design and implement youth sports programs that encompass the essential program features that foster PYD outcomes. Findings also underscore the need for ongoing coach education that reinforces the importance of building strong relationships with youth and encouraging youth engagement in sports.

Program quality assessment continues to be a central theme in youth development efforts. Youth participation and engagement in a quality sports program stimulate interest for social change and transformation. This study is important to help individuals and systems to make sound decisions about what assessment tools and procedures that will best meet program assessment and improvement. In particular, this study supports youth sports program administrators and coaches in assessing the quality of sports programs and linking this to developmental outcomes. Hence, leveraging them to establish the basis for a high-quality youth sports program, yielding developmental outcomes.

Exploring program quality and development outcomes related to a youth volleyball project in this sense provides insights from the perspectives of the players that will help to inform a positive development culture, emphasizing quality and equity at the same time. This exploration is particularly important for Ethiopia as it seeks to provide a broader dimension to its youth sports policies by utilizing the empirical evidence of relationships and variations to enhance national sporting excellence and promote a culture of development. Beyond this, the results have important practical implications for many youth volleyball programs and coaches who work with youth volleyball players.

## 5. Conclusions

Although youth sport has been considered an important mechanism for the attainment of PYD, it is considered a problematic area motivated by incentives associated with winning games rather than holistic youth development [7]. As such, youth players within these environments are often faced with challenges, which may hinder not only the development of sporting talent but also psychosocial development [44]. The findings in the current study provide evidence of attaining developmental outcomes within the Ethiopian youth sport context, facilitating the attainment of developmental outcomes relevant to both the youth players and the sport itself. However, there are still challenges that should be seriously considered as they mitigate further positive outcomes. The results of this study indicated that, for youth sport, positive athlete–coach relationships and a higher rate of practice were associated with the developmental outcomes measured. Contrary to these, challenges surrounding the implementation of the projects are significant threats that need attention as they disrupt the positive impacts of youth participation in sports. In general, the results suggest that youth volleyball coaches may be able to create suitable environments to deliver optimal youth sports programs, which foster positive developmental outcomes. However, there may still be prospects of better educating coaches with regards to the potential influence they have on young players, promoting the development of psychosocial skills far beyond a focus on talent identification and development in youth sports.

## Figures and Tables

**Table 1:** Demographic characteristics of participants.

Demographic Variables	Category	Count	Column *n* %
Gender	Male	107	49.8%
	Female	108	50.2%
Age	Male	M = 16.54	SD = 0.62
	Female	M = 15.81	SD = 0.76
Youth attendance	3 years	66	30.7%
	4 years	149	69.3%
Project site zone	Western zone	87	40.5%
	Central zone	64	29.8%
	Eastern zone	64	29.8%

**Table 2:** Descriptions of variables, measures, and definitions included in the study.

Variable Type	Measure	Definition
Independent variable	Gender	is represented by a dichotomous variable that takes on one of only two possible values when measured with the possible responses of female or male.
	Project site zone	refers to a cluster of project sites found in a similar geographic location.
Independent/Dependent Variable—Program Quality	Current practice	refers to the normative standards and procedures of the youth volleyball program as performed or applied by the program participants.
	Player’s experience	refers to all the activities a youth volleyball player engaged through while attending the program.
	Perceived challenge	refers to physical, emotional, cognitive difficulties or barriers perceived by the youth volleyball player in participating in the volleyball program.
	Coach–athlete relationship	refers to all situations in the youth volleyball program in which a coach and athlete’s emotional feelings, thoughts, and behaviors interact.
Dependent Variable—Developmental outcome	Perceived quality of talent identification and development system	refers to the youth volleyball players’ perceived level of quality in the youth development program in terms of physical health, education, and psycho-social development within youth athletes.
	Participation in youth volleyball	refers simply to the extent a youth volleyball player attends the youth volleyball program.
	Integrated system for youth volleyball	refers to the quality of the youth volleyball development system in terms of its alignment and balance within and between the parts.
	National and regional volleyball competitions	refers to the extent of having a schedule that provides youth volleyball players the opportunity to compete against other similar teams or clubs at the regional and national levels.

Note: In this study, the four program quality domains were used as the dependent variable during the variance test to compare group differences. Also, they were used as independent variables or predictor variables in measuring relationships using multiple regression analysis tests.

**Table 3:** Summary of the program quality and developmental outcome indicators/measures.

Indicator/Measure—No. of Items (Cronbach Alpha)	Item	Source
Current practice—19 items (0.87)	Volleyball coaches involved in youth training strictly follow and apply scientific training principles.	Authors’ construction
Player’s experience—11 items (0.87)	I think the community (players, parents, school community. etc.) feels happy about the participation of youth players in the volleyball project.	Authors’ construction
Perceived challenge—10 items (0.85)	Lack of sufficient training and coaching materials used for trainees and coaches in youth volleyball.	
Coach–athlete relationship—11 items (0.80)	I respect my coach.	Coach–Athlete Relationship Questionnaire CARQ [55]
Perceived quality of talent identification and development system—11 items (0.79)	There is an effective system for the identification of young talented athletes, so that the maximum number of potential top-level athletes is reached at the right time/age.	sport policy factors leading to international sporting success” (SPLISS) [56].
Participation in youth volleyball—7 items (0.82)	Adolescents and youths have opportunities to participate in volleyball at school, during physical education or extra-curricular activities.	
Integrated system for youth volleyball—10 items (0.87)	There is a well-structured national and regional system for youth volleyball development.	
National and regional volleyball competitions—7 items (0.87)	The national volleyball competition has relatively high standard compared with the international standards.	SPLISS [56].

**Table 4:** Results of t-tests for the quality of youth volleyball by Gender.

Variables	Gender	*n*	M	SD	t	Df	Effect Size Cohen’s d	95% Confidence Interval of the Difference	
								Lower	Lower
Perceived experience in youth volleyball	Male	107	2.58	0.318	−2.547	203.65	0.35 *	−0.178	−0.023
	Female	108	2.68	0.258					

Significance levels: * *p* < 0.05.

**Table 5:** Summary of descriptive statistics for program quality and developmental outcomes measured across project site zones.

Variables	Category	*N*	Mean	SD	Std. Error	95% Confidence Interval for Mean		Min	Max
						Lower Bound	Upper Bound		
Quality of talent identification & development system	Western zone	66	2.92	0.442	0.054	2.81	3.03	1.82	4.00
	Central zone	85	2.82	0.512	0.055	2.71	2.93	1.82	5.00
	Eastern zone	64	2.83	0.428	0.053	2.72	2.94	1.91	3.73
	Total	215	2.85	0.467	0.031	2.79	2.91	1.82	5.00
Participation in youth volleyball	Western zone	66	2.98	0.570	0.070	2.84	3.12	2.00	5.00
	Central zone	85	2.82	0.388	0.042	2.74	2.90	2.14	4.00
	Eastern zone	64	2.87	0.486	0.060	2.75	2.99	2.00	5.00
	Total	215	2.89	0.482	0.032	2.82	2.95	2.00	5.00
Integrated system for youth volleyball	Western zone	66	2.95	0.487	0.060	2.83	3.07	2.00	5.00
	Central zone	85	2.80	0.389	0.042	2.71	2.88	1.00	4.00
	Eastern zone	64	2.77	0.371	0.046	2.68	2.86	1.90	3.40
	Total	215	2.84	0.422	0.028	2.78	2.89	1.00	5.00
National and regional volleyball competitions	Western zone	66	2.88	0.560	0.069	2.74	3.02	1.86	5.00
	Central zone	85	2.81	0.445	0.048	2.71	2.90	1.71	3.71
	Eastern zone	64	2.69	0.444	0.055	2.57	2.80	1.57	3.57
	Total	215	2.79	0.487	0.033	2.73	2.86	1.57	5.00
Current practice of youth volleyball	Western zone	66	2.64	0.505	0.062	2.52	2.77	1.63	3.42
	Central zone	85	2.38	0.465	0.050	2.28	2.48	1.42	3.21
	Eastern zone	64	2.60	0.248	0.031	2.54	2.67	2.11	3.05
	Total	215	2.53	0.441	0.030	2.47	2.59	1.42	3.42
Perceived experience in Youth volleyball	Western zone	66	2.56	0.314	0.038	2.48	2.64	1.91	3.55
	Central zone	85	2.68	0.290	0.031	2.62	2.75	1.91	3.27
	Eastern zone	64	2.63	0.261	0.032	2.56	2.70	2.18	3.18
	Total	215	2.63	0.293	0.019	2.59	2.67	1.91	3.55
Perceived challenges in youth volleyball	Western zone	66	3.17	0.694	0.085	3.00	3.34	2.08	4.50
	Central zone	85	3.50	0.696	0.075	3.35	3.65	2.33	4.67
	Eastern zone	64	3.48	0.544	0.068	3.35	3.62	2.25	4.25
	Total	215	3.39	0.668	0.045	3.30	3.48	2.08	4.67
Coach athlete relationship in youth volleyball	Western zone	66	2.94	0.918	0.113	2.72	3.17	1.67	4.56
	Central zone	85	3.43	0.798	0.086	3.26	3.60	2.03	4.89
	Eastern zone	64	3.01	0.619	0.077	2.85	3.16	1.89	4.42
	Total	215	3.15	0.818	0.055	3.04	3.26	1.67	4.89

**Table 6:** Summary results of one-way ANOVA for the program quality variables between project site zones.

Variables	Category	Sum of Squares	df	Eta Square	F	Sig.
Current practice in youth volleyball	Between Groups	7.022	2	3.511	21.41	0.000
	Within Groups	34.769	212	0.164		
	Total	41.791	214			
Perceived experience in youth volleyball	Between Groups	2.384	2	1.192	15.79	0.000
	Within Groups	16.004	212	0.075		
	Total	18.387	214			
perceived challenges in youth volleyball	Between Groups	4.992	2	2.496	5.84	0.003
	Within Groups	90.677	212	0.428		
	Total	95.669	214			
Coach–athlete relationship in youth volleyball	Between Groups	0.118	2	0.059	8.22	0.000
	Within Groups	1.526	212	0.007		
	Total	1.644	214			

Note: df represents degree of freedom.

**Table 7:** Results of post hoc tests for the program quality and developmental outcomes measured across project site zones.

Dependent Variable	(I) Project Site Zone	(J) Project Site Zone	M D (I–J)	Std. Error	Sig.	95% CI	
						LB	UB
Current practice of youth volleyball	Western zone	Central zone	0.26442 *	0.08007	0.003	0.0747	0.4542
		Eastern zone	0.03977	0.06951	0.835	−0.1257	0.2053
	Central zone	Western zone	−0.26442 *	0.08007	0.003	−0.4542	−0.0747
		Eastern zones	−0.22465 *	0.05928	0.001	−0.3651	−0.0842
	Eastern zones	Western zone	−0.03977	0.06951	0.835	−0.2053	0.1257
		Central zone	0.22465 *	0.05928	0.001	0.0842	0.3651
Perceived experience in Youth volleyball	Western zone	Central zone	−0.12541 *	0.04754	0.024	−0.2376	−0.0132
		Eastern zone	−0.07158	0.05084	0.339	−0.1916	0.0484
	Central zone	Western zone	0.12541 *	0.04754	0.024	0.0132	0.2376
		Eastern zone	0.05383	0.04796	0.501	−0.0594	0.1670
	Eastern zones	Western zone	0.07158	0.05084	0.339	−0.0484	0.1916
		Central zone	−0.05383	0.04796	0.501	−0.1670	0.0594
Perceived challenges in youth volleyball	Western zone	Central zone	−0.33220 *	0.11411	0.012	−0.6025	−0.0619
		Eastern zone	−0.31656 *	0.10933	0.012	−0.5759	−0.0572
	Central zone	Western zone	0.33220 *	0.11411	0.012	0.0619	0.6025
		Eastern zone	0.01564	0.10169	0.987	−0.2251	0.2564
	Eastern zone	Western zone	0.31656 *	0.10933	0.012	0.0572	0.5759
		Central zone	−0.01564	0.10169	0.987	−0.2564	0.2251
Coach athlete relationship in youth volleyball	Western zone	Central zone	−0.48940*	0.14238	0.002	−0.8270	−0.1518
		Eastern zone	−0.06647	0.13699	0.878	−0.3918	0.2588
	Central zone	Western zone	0.48940 *	0.14238	0.002	0.1518	0.8270
		Eastern zone	0.42293 *	0.11618	0.001	0.1479	0.6980
	Eastern zone	Western zone	0.06647	0.13699	0.878	−0.2588	0.3918
		Central zone	−0.42293 *	0.11618	0.001	−0.6980	−0.1479

*. The mean difference is significant at the 0.05 level.

**Table 8:** Summary of total intercorrelations between program quality and developmental outcome variables.

Variables	M	SD	1	2	3	4	5	6	7
1. Quality of talent identification & development system	2.86	0.47	1						
2. Youth volleyball sport participation	2.79	0.57	0.51 **	1					
3. Integrated system for youth volleyball	2.83	0.51	0.46 **	0.42 **	1				
4. National & regional volleyball competitions	2.79	0.49	0.29 **	0.31 **	0.31 **	1			
5. Current practice in youth volleyball	2.53	0.44	0.51 **	0.47 **	0.52 **	0.36 **	1		
6. Perceived experience in youth volleyball	2.63	0.29	0.04	0.07	0.13	0.02	0.18 **	1	
7. Perceived challenges in youth volleyball	3.39	0.67	−0.16 *	−0.14 *	−0.19 **	−0.22 **	−0.18 **	−0.03	1
8. Coach–athlete relationship in volleyball	3.16	0.82	0.51 **	0.47 **	0.53 **	0.39 **	0.79 **	0.11	−0.13

Significance levels: * *p* < 0.05 and ** *p* < 0.01.

**Table 9:** Summary of multiple regression models for developmental outcomes predictions.

Variable	B	T	*SE*	*p*	*Beta*	F	R^2^
Quality of talent identification and development							
Current practice in youth volleyball	0.30	2.88	0.10	0.00	0.28 **	21.90 ***	0.294
Perceived experience in youth volleyball	−0.07	−0.74	0.09	0.46	−0.04		
Perceived challenges in youth volleyball	−0.05	−1.23	0.04	0.22	−0.07		
Coach–athlete relationship in youth volleyball	1.51	2.97	0.51	0.00	0.28 **		
Youth volleyball sport participation							
Current practice in youth volleyball	0.32	2.50	0.13	0.01	0.25 *	17.18 ***	0.247
Perceived experience in youth volleyball	−0.02	−0.17	0.12	0.87	−0.01		
Perceived challenges in youth volleyball	−0.06	−1.07	0.05	0.29	−0.07		
Coach–athlete relationship in youth volleyball	1.71	2.66	0.64	0.01	0.26 **		
Integrated system for youth volleyball							
Current practice in youth volleyball	0.27	2.42	0.11	0.02	0.23 *	24.47 ***	0.318
Perceived experience in youth volleyball	0.08	0.79	0.10	0.43	0.05		
Perceived challenges in youth volleyball	−0.08	−1.84	0.05	0.07	−0.11		
Coach–athlete relationship in youth volleyball	1.92	3.50	0.55	0.00	0.33 **		
Quality of National and regional volleyball competitions							
Current practice in youth volleyball	0.10	0.87	0.11	0.39	0.09	12.21 ***	0.189
Perceived experience in youth volleyball	−0.05	−0.51	0.11	0.61	−0.03		
Perceived challenges in youth volleyball	−0.12	−2.68	0.05	0.01	−0.17 **		
Coach–athlete relationship in youth volleyball	1.69	2.99	0.57	0.00	0.305 **		

Significance levels: * *p* < 0.05. ** *p* < 0.01. *** *p* < 0.001.

## Data Availability

The data that support the findings of this study are available on request from the corresponding author.

## References

[1] UNICEF (2019). Getting into the Game Understanding: The Evidence for Child-Focused Sport for Development.

[2] Burnett C. (2015). The ‘uptake’ of a sport-for-development programme in South Africa. Sport Educ. Soc..

[3] Bonell C., Hinds K., Dickson K., Thomas J., Fletcher A., Murphy S., Melendez-Torres G.J., Bonell C., Campbell R. (2016). What is positive youth development and how might it reduce substance use and violence?. A systematic review and synthesis of theoretical literature. BMC Public Health.

[4] Tadesse T., Asmamaw A., Habtemariam S., Mack D. (2018). Proposing and Testing Models for Assessing Student Engagement, Self-Regulation and Psychological Need Satisfaction in Ethiopian Sports Academy Setting. Sport J..

[5] Gano-Overway L.A., Newton M., Magyar T.M., Fry M.D., Kim M.S., Guivernau M.R. (2009). Influence of Caring Youth Sport Contexts on Efficacy-Related Beliefs and Social Behaviors. Dev. Psychol..

[6] Whitley M.A., Massey W.V., Camiré M., Boutet M., Borbee A. (2019). Sport-based youth development interventions in the United States: A systematic review. BMC Public Health.

[7] Armour K., Sandford R., Duncombe R. (2013). Positive youth development and physical activity/sport interventions: Mechanisms leading to sustained impact. Phys. Educ. Sport Pedagog..

[8] Claver F., Jiménez R., Gil-Arias A., Moreno A., Moreno M.P. (2017). The Cognitive and Motivation Intervention Program in Youth Female Volleyball Players. J. Hum. Kinet..

[9] Standage M., Vallerand R.J. (2014). Motivation in sport and exercise groups: A self-determination theory perspective. Group Dynamics in Exercise and Sport Psychology.

[10] Maleka E.N. (2017). Monitoring and evaluation of sport-based HIV/AIDS awareness programmes: Strengthening outcome indicators. SAHARA J..

[11] Levermore R. (2011). Evaluating sport-for-development: Approaches and critical issues. Prog. Dev. Stud..

[12] Roth J.L., Brooks-Gunn J. (2016). Evaluating youth development programs: Progress and promise. Appl. Dev. Sci..

[13] Côté J., Mallett C. (2013). Review of Junior Sport Framework Briefing Paper: Positive Youth Development through Sport.

[14] Strachan L., Côté J., Deakin J. (2011). A new view: Exploring positive youth development in elite sport contexts. Qual. Res. Sport Exerc. Health.

[15] Yohalem N., Wilson-Ahlstrom A., Fischer S., Shinn M. (2007). Measuring Youth Program Quality: A Guide to Assessment Tools.

[16] Fitch N., Ma’ayah F., Harms C., Guilfoyle A. (2017). Sport, Educational Engagement and Positive Youth Development: Reflections of Aboriginal Former Youth Sports Participants. Aust. J. Indig. Educ..

[17] Bean C., Kramers S., Camiré M., Fraser-Thomas J., Forneris T. (2018). Development of an observational measure assessing program quality processes in youth sport. Cogent Soc. Sci..

[18] Gratton C., Jones I. (2010). Research Methods for Sports Studies.

[19] Silliman B., Schumm W.R. (2013). Youth program quality survey: Youth assessment of program quality. Marriage Fam. Rev..

[20] Tadesse T., Asmamaw A., Habtemariam S., Edo B. (2020). Sports Academy as an Avenue for Psychosocial Development and Satisfaction of Youth Athlete’s in Ethiopian Sports Academies. Sustainability.

[21] Eime R.M., Young J.A., Harvey J.T., Charity M.J., Payne W.R. (2013). A systematic review of the psychological and social benefits of participation in sport for children and adolescents: Informing development of a conceptual model of health through sport. Int. J. Behav. Nutr. Phys. Act..

[22] Coutinho P., Mesquita I., Fonseca A.M., Côte J. (2015). Expertise Development in Volleyball: The Role of Early Sport Activities and Players’ Age and Height. Kinseology.

[23] Whitley M.A., Massey W.V., Camiré M., Blom L.C., Chawansky M., Forde S., Boutet M., Borbee A., Darnell S.C. (2019). A systematic review of sport for development interventions across six global cities. Sport Manag. Rev..

[24] Hodge K., Danish S., Forneris T., Miles A. (2016). Life skills and basic psychological needs: A conceptual framework for life skills interventions. Positive Youth Development through Sport.

[25] Gould D., Flett R., Lauer L. (2012). The relationship between psychosocial developmental and the sports climate experienced by underserved youth. Psychol. Sport Exerc..

[26] Gould D., Carson S. (2011). Young athletes perceptions of the relationship between coaching behaviors and developmental experiences. Int. J. Coach. Sci..

[27] Ryan R.M., Deci E.L. (2000). Self-Determination Theory and the Facilitation of Intrinsic Motivation, Social Development, and Well-Being. Am. Psychol..

[28] Bean C., Harlow M., Mosher A., Fraser-Thomas J., Forneris T. (2018). Assessing differences in athlete-reported outcomes between high and low-quality youth sport programs. J. Appl. Sport Psychol..

[29] Cranmer G.A., Sollitto M. (2015). Sport Support: Received Social Support as a Predictor of Athlete Satisfaction. Commun. Res. Rep..

[30] Jowett S. (2017). Coaching effectiveness: The coach–athlete relationship at its heart. Curr. Opin. Psychol..

[31] Ullrich-French S., McDonough M.H. (2012). Correlates of long-term participation in a physical activity-based positive youth development program for low-income youth: Sustained involvement and psychosocial outcomes. J. Adolesc..

[32] Super S., Hermens N., Verkooijen K., Koelen M. (2018). Examining the relationship between sports participation and youth developmental outcomes for socially vulnerable youth. BMC Public Health.

[33] Dawes N.P., Vest A., Simpkins S. (2014). Youth Participation in Organized and Informal Sports Activities Across Childhood and Adolescence: Exploring the Relationships of Motivational Beliefs, Developmental Stage and Gender. J. Youth Adolesc..

[34] Cairney J., Clark H.J., Kwan M.Y., Bruner M., Tamminen K. (2018). Measuring sport experiences in children and youth to better understand the impact of sport on health and positive youth development: Designing a brief measure for population health surveys. BMC Public Health.

[35] Holt N.L., Neely K.C. (2011). Positive youth development through sport: A review. Rev. Iberoam. Psicol. Ejerc. Deporte.

[36] De Bosscher V., De Knop P., Van Bottenburg M., Shibli S., Bingham J. (2009). Explaining international sporting success: An international comparison of elite sport systems and policies in six countries. Sport Manag. Rev..

[37] Henriksen K., Larsen C.H., Christensen M.K. (2014). Looking at success from its opposite pole: The case of a talent development golf environment in Denmark. Int. J. Sport Exerc. Psychol..

[38] Bean C., Forneris T. (2016). Examining the Importance of Intentionally Structuring the Youth Sport Context to Facilitate Positive Youth Development. J. Appl. Sport Psychol..

[39] Super S., Wentink C.Q., Verkooijen K.T., Koelen M.A. (2017). Exploring the Sports Experiences of Socially Vulnerable Youth. Social Inclusion..

[40] Wolde B., Gaudin B. The institutional organization of Ethiopian athletics. In Annales d’Ethiopie; Editions: Persée-Portail des Revues Scientifiques en SHS; 2007; Volume 23, pp. 471–493.

[41] Wilber R.L., Pitsiladis Y.P. (2012). Kenyan and Ethiopian distance runners: What makes them so good?. Int. J. Sports Physiol. Perform..

[42] Lerner R.M., Snyder C.R., Lopez S.J. (2009). The positive youth development perspective: Theoretical and empirical bases of a strength-based approach to adolescent development. The Oxford Handbook of Positive Psychology.

[43] Adom-Aboagye N.A.A., De Coning C., Bitugu B.B., Keim M. (2016). Trends and tendencies in the facilitation and training of sport and development programmes for the youth: Lessons of experience from African cases: Sport for development. Afr. J. Phys. Act. Health Sci..

[44] Harwood C., Johnston J. (2016). Positive youth development and talent development: Is there a best of both worlds?. Positive Youth Development through Sport.

[45] Yohalem N., Wilson-Ahlstrom A. (2010). Inside the black box: Assessing and improving quality in youth programs. Am. J. Community Psychol..

[46] Jacobs J.M., Wright P.M. (2018). Transfer of Life Skills in Sport-Based Youth Development Programs: A Conceptual Framework Bridging Learning to Application. Quest.

[47] Schulenkorf N., Sherry E., Rowe K. (2016). Sport for Development: An Integrated Literature Review. J. Sport Manag..

[48] Svensson P.G., Hancock M.G., Hums M.A. (2016). Examining the educative aims and practices of decision-makers in sport for development and peace organizations. Sport Educ. Soc..

[49] Bean C., Forneris T., Brunet J. (2016). Investigating discrepancies in program quality related to youth volleyball athletes’ needs support. Psychol. Sport Exerc..

[50] Ciocanel O., Power K., Eriksen A., Gillings K. (2017). Effectiveness of Positive Youth Development Interventions: A Meta-Analysis of Randomized Controlled Trials. J. Youth Adolesc..

[51] EFDRE (1998). Sport Policy of the Federal Democratic Republic of Ethiopia.

[52] Creswell J. (2012). Educational Research: Planning, Conducting, and Evaluating Quantitative and Qualitative Research.

[53] Taylor I.M., Bruner M.W. (2012). The social environment and developmental experiences in elite youth soccer. Psychol. Sport Exerc..

[54] Riley A., Anderson-Butcher D., Logan J., Newman T.J., Davis J. (2017). Staff practices and social skill outcomes in a sport-based youth program. J. Appl. Sport Psychol..

[55] Jowett S., Ntoumanis N. (2004). The Coach-Athlete Relationship Questionnaire (CART-Q): Development and initial validation. Scand. J. Med. Sci. Sports.

[56] De Bosscher V., Shibli S., Westerbeek H., van Bottenburg M. (2015). Successful Elite Sport Policies An International Comparison of the Sports Policy Factors Leading to International Sporting Success (SPLISS 2.0) in 15 Nations.

[57] Field A.P. (2009). Discovering Statistics Using SPSS: And Sex and Drugs and Rock ‘n’ Roll.

[58] Cohen J. (1988). Statistical Power Analysis for the Behavioral Sciences.

[59] Lenhard W., Lenhard A. (2016). Calculation of Effect Sizes.

[60] Cohen L., Manion L. (1994). Research Methods in Education.

